# Does genetic anticipation occur in familial Alexander disease?

**DOI:** 10.1007/s10048-021-00642-9

**Published:** 2021-05-28

**Authors:** Camille K. Hunt, Ahmad Al Khleifat, Ella Burchill, Joerg Ederle, Ammar Al-Chalabi, Jemeen Sreedharan

**Affiliations:** 1 Department of Basic and Clinical Neuroscience, King’s College London, London, UK; 2 Department of Neuroradiology, King’s College Hospital NHS Trust, London, UK

**Keywords:** Alexander disease, GFAP, Anticipation, Mosaicism

## Abstract

Alexander Disease (AxD) is a rare leukodystrophy caused by missense mutations of glial fibrillary acidic protein (*GFAP*). Primarily seen in infants and juveniles, it can present in adulthood. We report a family with inherited AxD in which the mother presented with symptoms many years after her daughter. We reviewed the age of onset in all published cases of familial AxD and found that 32 of 34 instances of parent–offspring pairs demonstrated an earlier age of onset in offspring compared to the parent. We suggest that genetic anticipation occurs in familial AxD and speculate that genetic mosaicism could explain this phenomenon.

## Case history

The proband had no prior medical history and normal early development. She presented to the neurology department aged 45 with an 8-month history of stiffness in all four limbs and poor balance. Despite this, she was running a café independently. There were no sphincter or sensory disturbances. Examination revealed poorly responsive pupils, a prominent jaw jerk and slight dysarthria. She had slow, spastic speech and slow tongue movements with no tongue wasting. She had marked spasticity in all limbs with brisk reflexes, which was slightly worse on the left, with normal strength except for the left first dorsal interosseous muscle (MRC grade 4). Her plantar reflexes were equivocal. There were no lower motor neuron features, no cerebellar signs and sensory examination was normal.

MRI scans of her brain and cervical spine were conducted at her local hospital and reported as normal. Spinal fluid demonstrated normal cellular constituents, normal glucose and CSF protein and no oligoclonal bands. Clinical neurophysiology assessments demonstrated normal nerve conduction and electromyography. However, central motor conduction times to intrinsic hand and foot muscles were markedly increased. Collectively, these findings were consistent with a progressive, degenerative purely upper motor neuron syndrome affecting limbs and with a pseudobulbar component. A presumptive diagnosis of primary lateral sclerosis was made and baclofen was commenced to treat spasticity. Her condition progressively deteriorated, impairing her mobility such that she had to retire from work. Six years after symptom onset, aged 51, she remained free of lower motor neuron signs. She was then lost to follow-up.

**Fig. 1:**
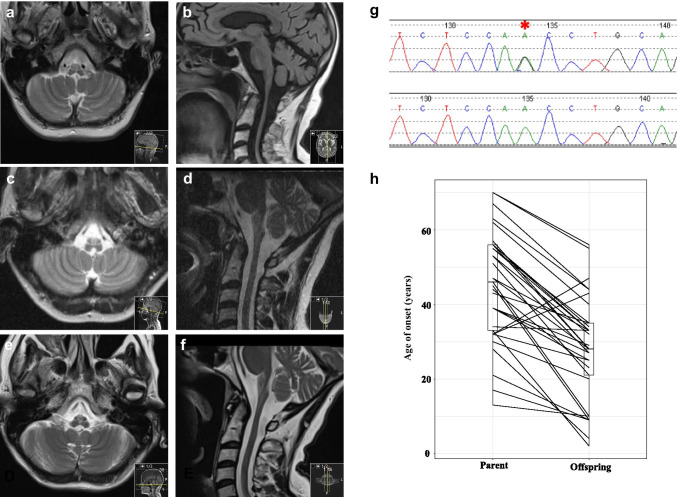
**a**–**f** MRI scans of proband and mother. Axial T2 (**a**) and parasagittal FLAIR (**b**) of proband’s mother at the time of presentation showing upper cervical spinal cord and medullary volume loss and demyelination. Axial T2 (**c**) and sagittal T2 (**d**) of proband at initial presentation and at the time of re-call to clinic (**e**, **f**). **g** DNA chromatograms demonstrating proband’s DNA sequence (top, *GFAP* mutation highlighted with asterisk) and a control DNA sequence. **h** Paired boxplot comparing age of onset between 34 parents and 34 offspring with familial AxD. Lines between boxplots represent individual parent–offspring pairs. Boxplot bars indicate 95% confidence intervals. PairedData package was used to generate the effect plot on R. Paired sample *t* test showed a mean 14% decrease in age of onset in the offspring group compared to parents (*p* = 2.02 × 10^–9^, 95% CI for the difference 12.64–20.93 years)

Three years after the proband’s last assessment, her 71-year-old mother presented to our clinic with a history strikingly reminiscent of her daughter’s ailments. The patient described weakness progressing over 4 years with recent dysphagia. At presentation she was wheelchair dependent. Examination demonstrated extreme weight loss, prominent palmomental reflexes, a bovine cough and spastic, dysarthric speech. No muscle wasting or fasciculations were observed in cranial or limb territories. Tone was increased in all limbs, with a pyramidal pattern of weakness, MRC grade 4 in the proximal limb muscles. Deep tendon reflexes were brisk and plantars were extensor. Brain MRI demonstrated cervicomedullary atrophy (Fig. 1a, b) suggesting a diagnosis of adult-onset AxD. A DNA sample for *GFAP* mutation screening to confirm the diagnosis could not be obtained, as the patient developed a chest infection and died soon after her MRI scan. However, we reconnected with the proband who returned to clinic for review.

By now, aged 54, the proband was wheelchair dependent. She reported intermittent choking on food. On neurological examination, she was alert and oriented. The pupils were equal at 3 mm and slowly reactive to light. She had weakness of neck flexion, a weak cough, spastic speech and a brisk jaw jerk. In addition to marked spasticity in all four limbs, she had proximal weakness of grade 4 in both upper and lower limbs in a pyramidal pattern, and all deep tendon reflexes were brisk. There were no lower motor neuron signs and no sensory deficits.

She underwent a repeat MRI brain scan (Fig. 1e, f), which showed marked thinning of the medulla oblongata and cervical cord at the cervicomedullary junction. Increased T2 cord signal/FLAIR foci were noted in the medulla with global cerebral atrophy in excess for her age. These features were similar to those of her mother and consistent with adult-onset AxD. Her previous imaging was obtained for comparison and on review by a specialist neuroradiologist was concluded to be consistent with AxD (Fig. 1c, d). A DNA sample was obtained and sequencing showed heterozygosity for the *GFAP* mutation c.1157A > G (p.Asn386Ser) (Fig. 1g). This mutation occurs in the C-terminal tail domain of GFAP and is predicted to be deleterious, with a combined annotation-dependent depletion score of 23.3 (CADD v1.6). The same sequence change has been reported several times in patients with AxD [1, 2] but only once in the gnomAD database of controls. Ours is the first description of this mutation in a case of familial AxD.

## Analysis of familial Alexander disease

An intriguing aspect of this family is that the age of symptom onset in the mother was greater than in the daughter by over two decades. Variable expressivity occurs in AxD [3, 4], but here both mother and daughter had strikingly similar phenotypes. Whilst variable penetrance of the *GFAP* mutation could explain the daughter’s much earlier age of onset, another possibility is genetic anticipation. To investigate further, we reviewed all previously published cases of familial AxD, searching Pubmed using the criteria “alexander [title] disease [title]”. We identified 22 studies reporting 37 instances of inherited AxD (Table 1) [3, 3–25]. Data on age of onset was available for 34 parents (27 female: 7 male) and 34 offspring (17 female: 17 male). The mean age of onset in the affected parents was 43.8 years (95% CI 38.2–49.4 years), and in offspring, 27.4 years (95% CI 22.4–32.4 years) (Fig. 1h). Paired sample *t* test showed a mean 14% decrease of the age of onset in the offspring group compared to parents (*p* = 2.02 × 10^–9^, 95% CI for the difference 12.64–20.93 years). This suggests that genetic anticipation is influencing disease onset in AxD.

**Table 1:**
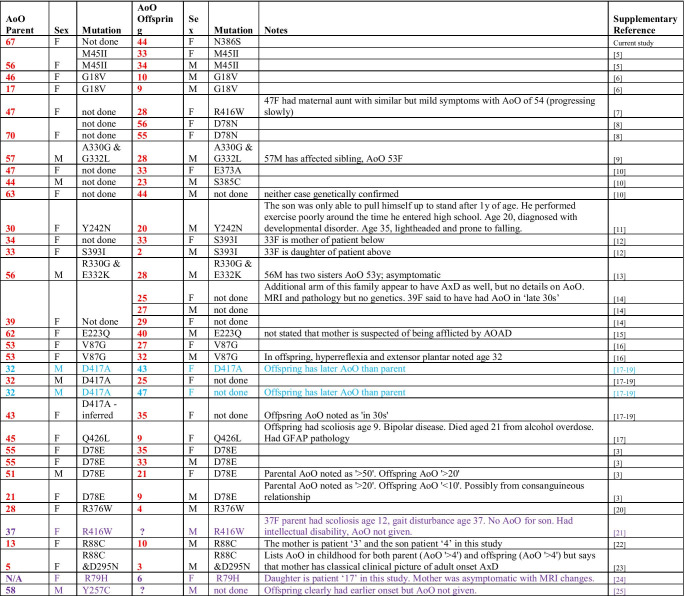
Summary of published cases of familial Alexander disease. Cases highlighted in blue text showed earlier age of onset in parent compared to offspring, whilst other cases demonstrated earlier age of onset in offspring. Purple cases were excluded from analysis as age of onset (actual or approximate) for either parent or offspring was not given or parent was asymptomatic. *AoO*, age of onset. *N/A* not applicable as asymptomatic

## Discussion

Genetic anticipation is a feature of neurological conditions linked to unstable nucleotide repeat motifs, such as Huntington’s disease. Genetic anticipation has also been described in polygenic disorders including Crohn’s disease and breast cancer, and in monogenic disorders such as von Hippel-Lindau syndrome. The mechanisms underlying apparent genetic anticipation in these conditions are unclear. Statistical artefacts such as ascertainment bias may be contributing factors, but these can be controlled for [26]. Indeed, in our family, this bias is highly unlikely given that the daughter was diagnosed before her mother had any symptoms. How genetic anticipation could occur in AxD, a disease caused largely by missense mutations, is unclear but there are several possibilities. For example, there may be a microsatellite repeat near the *GFAP* gene that influences its expression and expands during transmission, or epigenetic factors such as DNA methylation changes that influence *GFAP* expression. Alternatively, there may be linkage disequilibrium of the *GFAP* mutation with another variant that directly contributes to disease and gives rise to anticipation, such as a repeat expansion.

We postulate that another mechanism may explain apparent anticipation: genetic mosaicism. In AxD, most cases are caused by de novo mutations [27]. It is plausible that if an embryo developed a spontaneous *GFAP* mutation beyond the single-cell stage, it would be mosaic for this mutation, which could mean that only a fraction of the nervous system would express mutant *GFAP*. This could attenuate disease severity and delay the age of onset. If the mutation was present in the germ cells of this mosaic patient, it could be transmitted to their offspring, who would then express the mutation ubiquitously, resulting in a more severe phenotype and earlier age of onset. Interestingly, a person with AxD has been described in which a *GFAP* mutation was found to be present in cells taken from a cheek swab, but absent from DNA extracted from blood, indicating somatic mosaicism [28]. To our knowledge, mosaicism has not previously been suggested as a cause of apparent anticipation. We were unable to test this hypothesis in our family but suggest that this should be considered in future instances of inherited AxD. This would require sequencing of DNA extracted from different brain regions at post-mortem, or, where brain tissue is unavailable, comparison of peripheral tissues, such as skin, saliva and lymphocyte derived DNA.
